# Effectiveness of a mHealth platform-based lifestyle integrated multicomponent exercise (*PF-Life*) program to reverse pre-frailty in community-dwelling older adults: a randomized controlled trial study protocol

**DOI:** 10.3389/fpubh.2024.1389297

**Published:** 2024-06-07

**Authors:** Na Li, Feng Huang, Nan Wang, Siyang Lin, Yin Yuan, Pengli Zhu

**Affiliations:** ^1^The Shengli Clinical Medical College of Fujian Medical University, Fuzhou, China; ^2^Department of Nursing, Fujian Provincial Hospital, Fuzhou, China; ^3^The School of Nursing, Fujian Medical University, Fuzhou, China; ^4^Fujian Provincial Institute of Clinical Geriatrics, Fuzhou, China; ^5^Fujian Provincial Center for Geriatrics, Fuzhou, China; ^6^Fujian Provincial Key Laboratory of Geriatrics, Fuzhou, China

**Keywords:** pre-frailty, mHealth platform, lifestyle-integrated exercise, older adults, protocol

## Abstract

**Background:**

Pre-frailty represents an ideal window of opportunity to potentially prevent frailty and disability. Early and effective interventions to delay or reverse pre-frailty are public health imperative. The present trial aims to evaluate the effectiveness and underlying mechanisms of mobile health (mHealth) platform-supported lifestyle-integrated multicomponent exercise (*PF-Life*) to reverse pre-frailty in community-dwelling older adults.

**Methods:**

This is an open-label, prospective, two-arm parallel randomized controlled trial with allocation concealment and outcome assessment blinding. We aim to recruit 140 pre-frail community-dwelling older adults who will be randomized into two groups. The control group will receive a health education program, while the intervention group will receive *PF-Life* training as planned for 1 year. The proportion of pre-frailty, functional performance (muscular strength, aerobic capacity, flexibility, and balance), body composition, and physical activity will be measured at pre-intervention, post-intervention, and 12-month follow-up. Inflammatory biomarkers will also be collected to explore the underlying mechanisms.

**Discussion:**

This is the first study to evaluate the effects of a novel digital lifestyle-integrated multicomponent exercise for pre-frail older people. The results of this trial will provide much-needed information on the short-and long-term effects of *PF-Life* based on functional performance and body composition. Meanwhile, inflammatory biomarkers and physical activity levels will be used to elucidate the underlying mechanisms of *PF-Life*. The findings from this trial will provide evidence for the effectiveness of lifestyle multicomponent exercise intervention supported by the mHealth platform that may reverse or even halt the onset of frailty.

**Clinical trial registration:**

https://www.chictr.org.cn/showproj.html?proj=176477, identifier ChiCTR2200063431.

## Background

Population aging and its higher demand for healthcare services have led to an increased sense of urgency to develop practical approaches to promote successful aging and prevent age-related diseases and progressive functional decline (1). Frailty is one of the most problematic manifestations of the aging population (2). It is an age-related clinical syndrome characterized by reduced reserve function of multiple physiologic systems; thus, frail older adults are vulnerable to adverse health outcomes (3, 4). The underlying mechanisms of frailty may have often been associated with inflammation and dysregulated energy metabolism. Chronic inflammation has been strongly linked to physical dysfunction and frailty (5). The measurement of inflammation provides a simple way to track the progression of frailty (6).

It is noteworthy that frailty is a dynamic and progressive process (7). Pre-frailty is an early state of frailty characterized by diminished balance, muscle strength, and mobility (8). Although physical dysfunction is not as severe as frailty, the prevalence of pre-frailty is much higher than that of frailty (46% vs. 12%) (9). Moreover, once frailty is clinically manifested, the descent trajectory of physical function is difficult to reverse, and the transition from pre-frailty to health is much more likely than from frailty to health (23% vs. 3%) (10–12). Therefore, pre-frailty represents an ideal window of opportunity to potentially prevent frailty and reduce healthcare costs. Early and effective interventions to delay or reverse pre-frailty are a public health imperative (7).

Regular exercise is the central axis for preventing pre-frailty (13). Multicomponent exercise (aerobic, resistance, and balance training) is the most effective type of exercise (14, 15). Despite the overwhelming evidence that multicomponent exercise is highly beneficial in improving the functional capacity of the pre-frail population, less than one-third of older people engage in exercise and they tend to stop within 6 months, leaving a clear gap between evidence and action (16, 17). Notably, regular exercise is not an easy task for most pre-frail individuals. There are several barriers to exercise, including poor exercise tolerance, inconvenient transportation, lack of time, skills, and motivation, and limited access to exercise resources and funding (18). Thus, integrating exercise into daily life may be an innovative approach to encourage pre-frail adults to adopt active lifestyles and change exercise behaviors (19, 20). The original Lifestyle-integrated Functional Exercise (LiFE) program — developed by Clemson in 2012 for the older person at risk of falls — has been shown to improve physical function, decrease disability, and promote habit formation (21). Contrary to other traditional home-based exercise programs, the LiFE program has demonstrated high adherence and motivation (22, 23). Incorporating multicomponent exercise into daily life may provide a practical option for pre-frail individuals to exercise whenever and wherever they prefer and address the issues of accessibility and cost. However, these home-based exercise programs also present challenges as they struggle to provide on-site supervision, feedback, and encouragement, all of which are crucial factors in improving long-term adherence and efficacy (24).

Therefore, exploring how technological innovation can support such an exercise program is imperative. Recent advances in digital technology, such as wearable sensors and smartphone applications, provide an opportunity to improve uptake and sustainability of exercise programs (25). For instance, objective physical activity (PA) monitoring using wearable sensors can be useful in developing motivational strategies to enhance daily activity (26). Particularly, mobile health (mHealth) platforms consisting of apps and web portals synchronized with wearable devices appear to be an effective way to enable self-management and remote monitoring of home-based exercise (27, 28). To date, little is known about the potential application of mHealth platforms to lifestyle-integrated multicomponent exercise and their effectiveness to prevent pre-frailty.

We have developed a lifestyle-integrated multicomponent exercise program, namely *PF-Life*, based on the mHealth platform in line with current evidence-based principles. This program offers tailored, progressive, lifestyle-integrated multicomponent exercises that incorporate behavioral change strategies such as timely motivation, feedback, reminders, and monitoring. The current randomized controlled trial (RCT) aims to evaluate the effectiveness and the underlying mechanisms of the *PF-Life* program to reverse pre-frailty in community-dwelling older adults. We hypothesize that *PF-Life* reverses pre-frailty, enhances functional fitness (FF), including endurance, flexibility, muscular strength, and balance, improves body composition (BC), increases daily PA, and modulates levels of inflammatory cytokines in a Chinese pre-frail population throughout a 12-month outcome assessment.

## Methods

### Study design and setting

**Figure 1:**
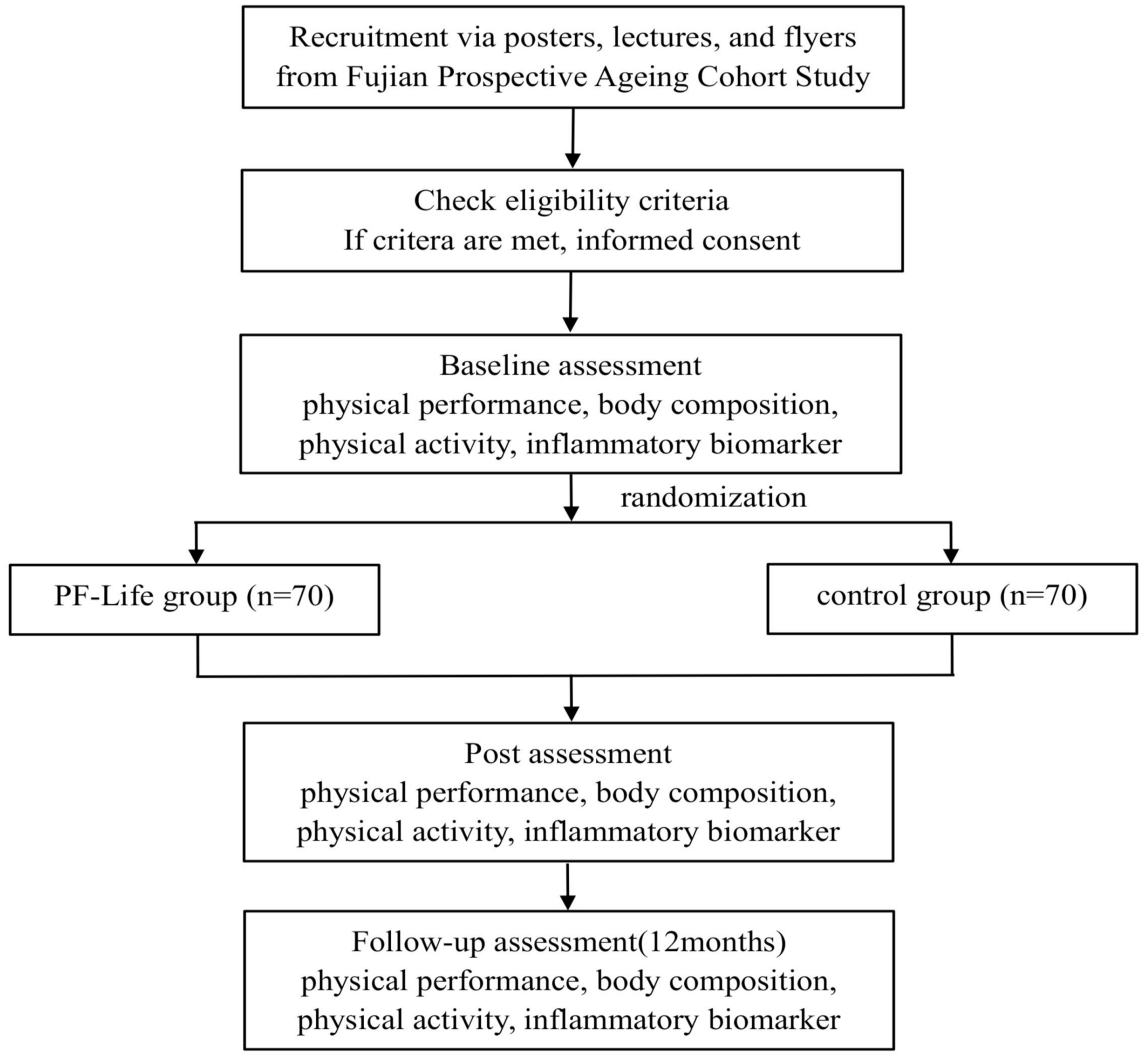
Flowchart of the study protocol.

This is an open-label, prospective, two-arm parallel RCT with balanced randomization (1:1), allocation concealment, and outcome assessment blinding. The study protocol employs the Standard Protocol Items: Recommendations for Interventional Trials (SPIRIT) guideline and the Consolidated Standards of Reporting Trials (CONSORT) statement for clinical trial transparency (29). The trial will be conducted at the Fujian Geriatrics Center, which is the largest geriatrics specialty among general hospitals in Fujian Province, China. Participants will be recruited from the *Fujian Prospective Aging Cohort* according to the screening criteria and randomly allocated to two groups: the *PF-Life* program (intervention) group and the health education (control) group. Data from each participant group will be collected at four different times: screening assessment, post-randomization baseline assessment, 4-month outcome assessment, and 12-month outcome assessment. The flowchart of the study protocol is displayed in Figure 1.

### Participants and recruitment

We aim to include 140 participants in this 1-year RCT. Recruitment will be carried out through posters, lectures, and flyers at the community health centers where the project team previously conducted the *Fujian Prospective Aging Cohort* Study. The cohort study was conducted in five community centers in Fuzhou, China, with a prevalence of pre-frailty of 44% (30). Participants inclusion criteria are: (1) aged ≥60 years; (2) living in the community; (3) fulfilling one or two of the Fried frailty phenotypes (31); (4) a smartphone user; (5) being able to walk independently without an assistive device; and (6) having normal language expression and comprehension (speaking Mandarin). Exclusion criteria are: (1) acute myocardial infarction within the past 3 months; (2) unstable or persistent cardiovascular or respiratory condition; (3) moderate to severe cognitive impairment, psychiatric disorders that prevent cooperation; (4) traumatic fracture (within 6 months); (5) performing moderate-to-vigorous PA more than 150 min per week during the past 3 months; and (6) inability to exercise or other conditions that may interfere with exercise.

### Sample size

The sample size is calculated using the statistical software PASS v11.0 (NCSS, Kaysville, UT, United States). Based on previous studies (32, 33), the proportion of reversing a pre-frailty state in the health education group and exercise groups is assumed to be 10 and 40%, respectively. Using a two-sided Fisher’s exact test and considering a dropout of approximately 20% (34, 35), the trial requires 118 participants (59 in each group, 50% each) to obtain 90% certainty (power = 1-*β*) of detecting a statistical difference at *α* = 0.05 (two-sided) level of significance. In order to increase the efficacy of the study, a total of 140 individuals will be recruited in this study (70 in each group).

### Randomization, blinding, and allocation concealment

To ensure allocation concealment, participants will be randomly assigned to the *PF-Life* group or control group in a 1:1 ratio by study staff (who will not be involved in participant recruitment or outcome assessment). The random number sequence will be generated by the investigator using a simple randomization procedure.^1^ These random numbers will then be individually placed in sequentially coded, sealed, opaque envelopes. The researcher will open the envelopes sequentially and assign random numbers to participants to identify their group. In addition, participants and interventionists will be instructed not to disclose the allocation to outcome assessors and data analysts. Unblinding will occur after all data analyses have been completed and will be carried out by staff not directly involved in the study.

### Intervention group

The exercise program will be delivered using the *PF-Life* mHealth platform, which is designed based on evidence from app developers, designers, geriatricians, and researchers to create personalized digital exercise programs. The mHealth platform consists of a smartphone app synchronized with a wearable device and a web-based portal that provides intensive interactions combined with multiple features and content. Based on Behavior Change Wheel (BCW) framework (36), 12 behavior change strategies, including goal setting, action plans, feedback, and motivation, will be applied in the development of *PF-Life* platform to support a lifestyle integrated exercise (37). The integration of these strategies with the *PF-Life* platform and interventions is described in Supplementary Table S1.

#### Introductory session

The first face-to-face introductory session will be held at the Fujian Geriatrics Center to introduce the *PF-Life* program, customize an exercise program based on functional performance assessment, discuss strategies and techniques for incorporating multicomponent exercise into daily lives, and guide users on how to place the wearable devices and download and use the app.

#### Exercise program

The exercise program is based on and expands upon the standardized LiFE program (21, 38). It is a multi-component exercise program that incorporates four basic functional areas: aerobic conditioning, muscle strengthening, flexibility, and balance training. When designing content, each exercise was specifically chosen to be relevant to daily living activities in the home environment and was easy to incorporate or mimic daily life, such as lifting the toes and heels, chair rise, back scratch, simulated towel wringing, farmer’s walk, etc.

There are 16 sets in the three levels of difficulty based on functional performance: elementary, intermediate, and advanced with increasing complexity. Personalized exercise programs are customized based on the assessment of the participant’s current level of FF in endurance, balance, flexibility, and strength. The exercise programs follow the principle of progression, increasing the exercise load by adding more repetitions and longer training times and using ankle weights at higher levels. For example, the first month of strength training begins with 3 reps per set for 3 sets at 30% one repetition maximum (1RM) loading and progresses in the second month with a gradual progression to 5 reps per set for 3 sets at 80% 1RM. Frequency, Intensity, Timing, and Type (FITT) are based on the FITT Guidelines for Older Adults (39). Participants are advised to train five times a week at their preferred time for 16 weeks. After 16 weeks, participants may continue to use the program for as long as they choose. In addition to the multicomponent exercises, participants are encouraged to increase daily PA and decrease sedentary time through visual charts showing daily PA progress. The exercise program was developed by the research team in consultation with experts in geriatrics, sports medicine, and rehabilitation medicine. The instructional videos were filmed in advance and embedded in the mHealth platform.

#### Implementation of the technology

Participants access the exercise program through the *PF-Life app*. The exercise tasks menu on the main interface present participants with a personalized list of exercises for each week. Tapping accesses the exercise task details, and participants perform the exercises while watching videos on the app. After completion of each exercise, feedback on the participant’s performance is captured on their smartphone and sent to healthcare professionals. Participants access visualized PA data uploaded by the wearable device, ActiGraph wGT3X-BT, as well as task completion, task progress bar, and exercise ranking, for self-monitoring. At the same time, participants use the app to communicate and interact with healthcare professionals via text, voice or video.

**Figure 2:**
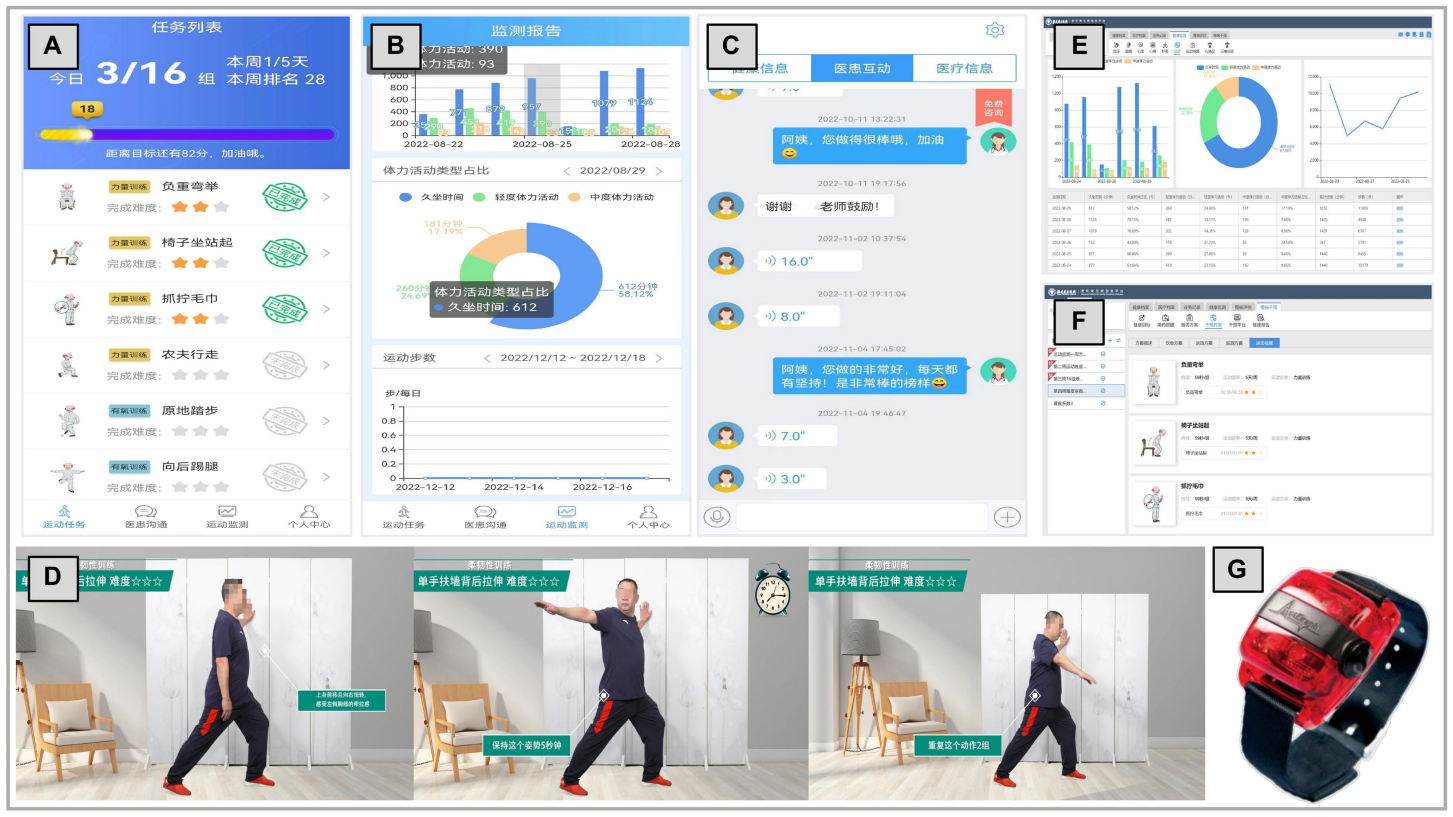
Screenshots of the representative function and wearable device: **(A)** personalized list of exercises for each week, **(B)** visualized physical activity data, **(C)** communication and interaction, **(D)** exercise video, **(E)** exercise monitor, **(F)** customize exercise program, and **(G)** ActiGraph wGT3X-BT.

The *PF-Life* web portal is designed for healthcare professionals. Healthcare professionals customize exercise programs for each participant through the exercise task features and exercise parameters, and other data are transferred directly to the participant’s smartphone. The web portal allows healthcare professionals to view data from participants’ wearable devices and smartphones, monitor participants’ exercise progress, and make timely adjustments. In addition, healthcare professionals are reminded to intervene when exercise tasks are not completed by sending reminders or motivational text messages. Exercise completion is automatically recorded by the *PF-Life* app during intervention and follow-up, and PA data are uploaded from the wearable device (ActiGraph wGT3X-BT) and synchronized with the *PF-Life* web portal. Healthcare professionals assess and record participants’ adherence to the exercise program by monitoring their physical activity data (the number of days exercised per week, number of sets exercised per day, and the duration of exercised per set). Figure 2 shows screenshots of the main application features and representative functions of the *PF-Life* Platform.

### Waitlist control group

The control group will maintain their regular lifestyle and daily routine activities. They will also receive four health education sessions once a month, delivered by geriatric specialist nurses in person or via WeChat, including the benefits of exercise for frailty. If the intervention proves effective for the intervention group, the control group will be invited to participate at the end of the study.

### Outcome measures

Table 1 provides a detailed summary of primary and secondary outcome measures for each assessment time point. Data collectors will be blinded to group assignments.

**Table 1:** Overview of primary and secondary outcomes, outcome measures, and assessment time points.

Outcomes	Outcome measures	Screening	Baseline	16 weeks	1-years
Eligibility assessment	Inclusion criteria	√			
	Exclusion criteria	√			
Primary outcomes					
Pre-frailty assessment	Fried frailty phenotype	√	√	√	√
Secondary outcomes					
Body composition	Body mass index, body fat mass, body fat percentage, fat-free body mass, fat-free mass percentage, and visceral fat level	√	√	√	√
Bone mineral density	Bone mineral density	√	√	√	√
Functional performance assessment	30-s chair rise test	√	√	√	√
	30-s arm curl test	√	√	√	√
	Hand grip strength	√	√	√	√
	Chair sit and reach test	√	√	√	√
	Back scratch test		√	√	√
	Timed up and go test		√	√	√
	Single-leg stance test		√	√	√
	4-m gait speed test		√	√	√
	2-min step test		√	√	√
Physical activity level assessment	Sedentary time (min/d), energy expenditure (MET/h), LPA (min/d), MVPA (min/d)		√	√	√
Inflammatory cytokines	Chemokines (CXCL8/IL-8, CCL5/RANTES, CXCL9/MIG, CCL2/MCP-1, CCL11/Eotaxin and CXCL10/IP-10, CCL4/MIP-1β, CCL7/MCP-3, CXCL1/GROα, CCL3/MIP-1α, CCL28/CTACK, and CXCL11/SDF-α)		√	√	√
	Cytokines (IL-2, IL-3, IL-4, IL-5, IL-6, IL-7, IL-9, IL-10, IL-12, IL-13, IL-15, IL-16, IL-17A, IL-18, IL-1β, TNF-a, TNF-β, IFNγ, and IFNα)		√	√	√
Exploitative outcome assessment	Medical and demographical data	√	√	√	√
	Sustainability		√	√	√

√, data will be obtained; MET, metabolic equivalents of task; LPA, low physical activity; MVPA, moderate-to-vigorous physical activity.

### Primary outcome assessment

The primary outcome is the proportion of participants with pre-frailty in the intervention group compared with the control group over 12 months from baseline, as measured by the Fried frailty phenotype, which is currently an internationally recognized standard for frailty identification and assessment. The frailty criteria have five components (29): (i) unintentional weight loss (>4.5 kg or at least 5% of body mass in the previous year through a self-reported questionnaire); (ii) low handgrip strength (HGS, cutoff points: ≤30 kg for males and ≤18 kg for females); (iii) fatigue [self-reported using questions 7 and 20 of the Centre for Epidemiological Studies Depression Scale (CES-D)]. Study participants are asked how often in the past week they think they have had difficulty completing daily tasks and how often they have been unable to complete activities. A positive response of ‘most of the time’ or ‘always’ to either question is considered an indication of fatigue; (iv) slow gait (≥0.65 m/s walking speed over a 4.5 m distance); and (v) low PA levels (<383 kcal/week for males and <270 kcal/week for females). The presence of one or two of the five components is recognized as pre-frailty.

### Secondary outcomes

Secondary outcomes are the change in BC, FF, bone mineral density (BMD), inflammatory cytokines, PA, and sedentary behaviors.

#### BC and BMD

The BC assessment will be performed according to the protocol described by Park et al. (40), using the Inbody 770 Body Composition Analyzer (CA, United States), which measures and analyses body mass index, body fat mass, body fat percentage, fat-free body mass, fat-free mass percentage, and visceral fat level (41). BMD of the heel bone in older people will be measured using a KJ 3000S+ ultrasound bone densitometer. The T-value is used to express the standard deviation of the participants’ BMD compared with the mean value of the same-sex peers. BMD is one of the most important methods for diagnosing osteoporosis, T ≤ −2.5SD indicates osteoporosis, T ≥ −1.0SD indicates normal bone mineral density, and −2.5SD < T < −1.0SD is considered bone loss (42, 43).

##### Functional performance assessment

Functional performance evaluates muscular strength (lower and upper body), aerobic capacity, flexibility, and balance.

###### Muscular strength

(1) 30-s chair rise test: this is a simple but reliable measure of lower extremity strength. It measures the number of times a participant can complete a rise and sit in 30 s without holding the chair (44, 45). (2) 30-s arm curl test: upper extremity strength is tested using the 30-s arm curl test. It measures the number of times the participant completes flexion-extension of the elbow in 30 s (46). (3) HGS: this is a fundamental indicator for determining frailty and disability. The left and right handgrip strength is measured alternately using the Jamar® Hydraulic Hand Dynamo meter (United States), three times each, and the maximum left/right HGS measurement is recorded (47, 48).

###### Flexibility

(1) Chair sit and reach test: the participant is seated on the edge of a chair without armrests, with the tested leg straight ahead at the knee, heel touching the ground, and dorsiflexion of the foot at a 90° angle, and the other leg naturally bent flat on the ground. The overlapping middle fingers of both hands are aligned as far as possible toward the toes. The distance between the tip of the middle finger and the tip of the foot is measured (49, 50). (2) Back scratch test: the participant stands upright with the dominant upper limb flexed at the shoulder and elbow, fingertips down, and palm inward to touch the center of the back. The other hand goes around the waist palm outward, fingertips upward with the back of the hand to touch the center of the back, and the hands are folded or touched as much as possible (51, 52).

###### Balance

(1) Timed up-and-go (TUG) test: it is useful for identifying older adults who may benefit from exercise interventions to reduce the risk of falls. The participant sits in a standard 45 cm highchair, gets up from the chair and then walks forward as quickly as possible for 3 m, and then returns to a sitting position; the time taken to complete the task is recorded, <10s is considered as normal (53). (2) Single-leg stance test: it is a significant predictor of injurious falls. Participants are instructed to balance on one leg without upper body support and the longest time standing on one leg is recorded (54).

###### Aerobic endurance

(1) 4-m gait speed test: the participant is instructed to walk a straight-line distance of 4 m on a flat surface at normal speed and the time taken to walk is recorded. Repeated measurement 2 time and the shortest time will be taken as the result of the test, a gait speed of <0.8 m/s was found to have good sensitivity and specificity for identifying frailty in old age (55, 56). (2) 2-min standing marching test: the participant is instructed to march in place, raise one knee above the height of the other knee, and count the number of times the right knee is raised (above the other knee) in 2 min (57, 58).

#### PA and sedentary behaviors

The PA characteristics will be measured using the ActiGraph wGT3X-BT activity monitor (ActiGraph, United States), which has been validated to measure PA intensity or activity energy expenditure in adults. The sampling frequency of ActiGraph wGT3X-BT will be set to 100 Hz, and participants will be instructed to wear the device 24 h a day for at least 7 days. Moderate-to-vigorous intensity PA levels [>3 metabolic equivalents of task (MET)] will be calculated using the Freedson cutoff point of ≥1952 counts/min (CPM), using the vertical axis (VA) in minutes/day. Sedentary behavior (<1.5 MET) will be calculated using a Freedson cutoff point of <100 CPM, using the VA in minutes/day, and the data will be processed using the ActiLife 6 software system (59). The wearable device automatically transmits to the healthcare professionals’ platform in real-time during the data collection process, thus validating its accuracy.

#### Inflammatory biomarkers

A registered nurse will collect approximately 5 mL of whole blood from the participants’ anterior elbow vein in the morning (after 12 h of fasting) into polypropylene tubes containing ethylenediaminetetraacetic acid (EDTA) and centrifuge the tubes at 4,000 rpm for 10 min at 4°C. All tubes will be collected, processed, and stored at −80°C in a refrigerator. Simultaneous measurement of 48 different inflammatory markers in plasma samples by Luminex assay according to previous literature (6), including serum levels of chemokines (CXCL8/IL-8, CCL5/RANTES, CXCL9/MIG, CCL2/MCP-1, CCL11/Eotaxin and CXCL10/IP-10, CCL4/MIP-1β, CCL7/MCP-3, CXCL1/GROα, CCL3/MIP-1α, CCL28/CTACK, and CXCL11/SDF-α) and cytokines (IL-2, IL-3, IL-4, IL-5, IL-6, IL-7, IL-9, IL-10, IL-12, IL-13, IL-15, IL-16, IL-17A, IL-18, IL-1β, TNF-a, TNF-β, IFNγ, and IFNα). Samples will be measured in duplicate.

#### Demographic and other baseline characteristics

Participants’ demographics (age and gender), socioeconomic attributes (marital status, education level, and average monthly income), lifestyle (smoking habits and alcohol consumption), and comorbidity will be collected through a structured questionnaire to prevent the influence of confounding factors such as demographics and other baseline variables on the results.

#### Data management

Participant’s information will be retained, except for their names, personal identities, or any other information that might reveal their identity. The results of the study and samples will be attached to a list using a special code. Only authorized researchers will be allowed to view the list. Data transferred to a dedicated server via the smartphone app will be encrypted. The dedicated server is installed in the Fujian Geriatrics Center.

### Statistical analysis

All data will be processed and analyzed using IBM SPSS 22.0 statistical analysis software, and a significance level of 5% will be used. Independent *t*-tests or *Mann–Whitney* tests will be used to compare demographic and other baseline characteristics of participants pre-intervention. A two-way ANOVA test with repeated measures for each of the variables will be applied to assess the effect of the program at pre-intervention, post-intervention, and follow-up stages on complexity (non-linear measures) vs. traditional (linear) measures of the proportion of pre-frailty, BC vs. BMD, PA vs. sedentary behaviors, FF (4-m gait speed test, TUG test, chair sit and reach test, back scratch test, single leg stance test, HGS, and 30-s chair rise test), and inflammatory cytokines. A subgroup analysis of the intervention group will be conducted after 12-month follow-up to explore the potential impact of continued exercise on the sustainability of the intervention effects. Multiple equation chain interpolation (MICE) is used to handle missing data, and where missing data are associated with observed covariates, propensity scores may be used to weight observations or adjust models to balance groups defined by missing data status. Statistical analyses will be performed using per-protocol and intent-to-treat (ITT) analyses in the case of potential dropouts.

## Discussion

Multicomponent exercise is the most effective strategy for preventing pre-frailty (13). The earlier the intervention, the greater the potential benefits. However, low adherence to engagement in traditional multicomponent exercises has led to an increased sense of urgency to design new alternatives. Applying the *PF-Life* program to the pre-frail older population has multiple advantages.

First, incorporating multicomponent exercises into daily life can help older adults form exercise habits by linking training to daily tasks. Second, the *PF-Life* program is flexible and easy to adopt, with exercise equipment derived from daily life, potentially eliminating time, resource, equipment, and transportation constraints required for exercise (60, 61). Third, the *PF-Life* program is designed to be individualized according to the FF level, which is conducive to long-term adherence (62). Finally, combining evidence-based exercise for frailty prevention with behavioral technology uses emerging technologies to provide personalized interventions, feedback, and motivation, and helps people track exercise outcomes.

To explore the underlying mechanisms, the effects of lifestyle-integrated multicomponent exercise on BC and FF in pre-frail older adults will be assessed. Multicomponent exercise has been found to reduce body fat loss, increase fat-free mass, and improve physical function in both healthy and frail older adults, suggesting that BC and physical dysfunction may serve as potential markers and modifiable risk factors for frailty (63, 64) and therefore may be a key target for treating pre-frail older adults. In addition, chronic low-grade inflammation is recognized as a key factor contributing to frailty and is a hallmark of aging. It has been found that higher levels of inflammatory mediators in older populations can directly influence the onset of frailty by degrading proteins or indirectly influencing metabolic pathways, leading to frailty (6). Levels of pro-inflammatory chemokines and cytokines such as interleukin-1β (IL-1β), interleukin-6 (IL-6) and tumor necrosis factor-α (TNF-α) are known to be elevated in frail individuals, whereas levels of the anti-inflammatory cytokines interleukin-4 (IL-4) or interleukin-10 (IL-10) are reduced (65). However, there is a lack of evidence on the beneficial effects of exercise interventions based on digital technology on inflammatory levels in pre-frail older adults, particularly in terms of inflammatory biomarkers. However, physical exercise can reduce certain inflammatory biomarkers in aging older adults. Since the balance between inflammatory and anti-inflammatory biomarkers is thought to be a determinant of the severity of aging-related diseases, understanding the effects of PA on the inflammatory profile of frail and aging older adults will provide insights into the mechanisms by which exercise provides benefits and guide future customization of exercise programs for frail older adults.

To the best of our knowledge, this is the first RCT to evaluate the effectiveness of a novel and innovative digital lifestyle-integrated multicomponent exercise program in reversing pre-frailty in community-dwelling older adults and shed light on the underlying inflammatory mechanisms from a scientific perspective. The trial will use rigorous methods to reduce biases, such as randomization, active recruitment strategy, rigorous eligibility screening, innovative design of mHealth technology based on behavioral change theory, blinding of outcome assessors and data analysts, and statistical analyses based on ITT principles.

## Limitations

It is important to note that the intervention we studied was specifically designed for older adults who use smartphones. While there has been a significant increase in smartphone usage among older adults in China, particularly due to COVID-19 prevention and control measures, this still limits the generalizability of our findings to a broader population of seniors. Nonetheless, research indicates that digital technology-based interventions may be more motivating, engaging, and appealing to older adults compared to traditional methods. Furthermore, such virtual exercises could serve as an effective alternative to in-person physical activities during times when outdoor activities are restricted due to unforeseen circumstances, such as mandatory closures related to COVID-19 or adverse weather conditions (66). In addition, participants and intervention staff could not be blinded due to the difficulty of blinding in an exercise intervention. It may increase the risk that the results of the study will be influenced by the expectations of the participants and the researcher.

## Ethics statement

Written informed consent was obtained from the individual(s) for the publication of any potentially identifiable images or data included in this article.

## Author contributions

NL: Data curation, Formal analysis, Investigation, Writing – original draft. FH: Supervision, Writing – review & editing. NW: Investigation, Writing – original draft. SL: Investigation, Writing – original draft. YY: Investigation, Writing – original draft. PZ: Funding acquisition, Methodology, Writing – review & editing.

## Footnotes

^1^ http://www.randomizer.org/
