# Supplementary material for: Effectiveness of a mHealth platform-based lifestyle integrated multicomponent exercise (PF-Life) program to reverse pre-frailty in community-dwelling older adults: a randomized controlled trial study protocol
Source: Front Public Health. 2024 Jun 7;12:1389297. doi: 10.3389/fpubh.2024.1389297 (PMC11190369; doi:10.3389/fpubh.2024.1389297)
Supplement: Supplementary file 1 [file Table_1.docx]

**Supplementary table1 Behavior change techniques and its corresponding platform functions**

| COM-B  components | Intervention functions | Behavior change techniques (BCTs) | *PF-Life* platform module |
| --- | --- | --- | --- |
| physical capability | Training, Education | Instruction on how to perform the behaviour  Demonstration of the behavior | Video tutorials for exercise execution |
| Psychological  capability | Training, Education,  Enablement | Instruction on how to perform a behaviour  Demonstration of the behavior | Video tutorials for exercise execution |
| Physical opportunity | Environmental,  Restructuring | Material incentive (behavioral) | Campaign rankings sharing |
| social opportunity | Environmental,  Restructuring,  Enablement | Restructuring the physical  environment | Video offers advice on how to incorporate exercise into life |
| Reflective motivation | Education,  Persuasion,  Enablement | Goal setting (behavior)  Goal setting (outcome)  Action planning  Review behavior goal  Feedback on behavior  Self-monitoring of behavioral  Habit formation | Set weekly activity goal  Exercise data monitoring  Exercise report feedback  Historical data access  View exercise progress  Track minutes of exercise  Export exercise data |
| Automatic motivation | Incentivization,  Education | Self-monitoring of behaviour  Prompts/cues  Material incentive (behavioral) | View exercise progress  Notifications are sent to users  when they miss their weekly goals  Campaign rankings sharing |
